# Observation of arenavirus nucleoprotein heptamer assembly

**DOI:** 10.1002/2211-5463.13106

**Published:** 2021-02-25

**Authors:** Nicolas Papageorgiou, Afroditi Vaitsopoulou, Awa Diop, Thi Hong Van Nguyen, Bruno Canard, Karine Alvarez, François Ferron

**Affiliations:** ^1^ Laboratoire Architecture et Fonction des Macromolécules Biologiques (AFMB) Aix‐Marseille University and CNRS France; ^2^ School of Life & Health Sciences Aston University Birmingham UK; ^3^ European Virus Bioinformatics Center Jena Germany

**Keywords:** *Arenaviridae*, nucleoprotein, assembly, *Bunyavirales*

## Abstract

Arenaviruses are enveloped viruses containing a segmented, negative, and ambisense single‐stranded RNA genome wrapped with a nucleoprotein (NP). The NP is the most abundant viral protein in infected cells and plays a critical role in both replication/transcription and virion assembly. The NP associates with RNA to form a ribonucleoprotein (RNP) complex, and this implies self‐assembly while the exact structure of this polymer is not yet known. Here, we report a measurement of the full‐length Mopeia virus NP by negative stain transmission electron microscopy. We observed RNP complex particles with diameter 15 ± 1 nm as well as symmetric circular heptamers of the same diameter, consistent with previous observations.

## Graphical abstract

The nucleoprotein associates with RNA to form a ribonucleoprotein complex. The nucleoprotein trends to self‐assemble into heptameric rings. Theses rings represent stable intermediate states representative of the average full turn of the Arenaviridae ribonucleoprotein filament. They are the first observation of the complexed nucleoprotein‐RNA as a polymer and hint that oligomerization occurs as it happens for other *Bunyavirales* nucleoproteins.

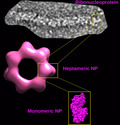

**Abbreviations**
IGR: intergenic region
LASV: Lassa virus
MACV: Machupo MACV
MOPV: Mopeia virus
mRNA: messenger RNA
NP: nucleoprotein
PICV: Pichinde virus
RNP: ribonucleoprotein
TEM: transmission electron microscopy
vRNA: viral RNA

Arenaviruses are zoonotic viruses that cause chronic infections in rodents which constitute a reservoir of human pathogens worldwide. *Arenaviridae* is part of the *Bunyavirales* order and regroups *Mammarenavirus*, *Reptarenavirus*, *Hartmanivirus*, and *Antennavirus* genera [1, 2]. *Mammarenaviruses* are further classified into two groups based on their geographic distribution and phylogeny: the Old World arenaviruses and the New World arenaviruses. Until now, all the human pathogens such as Lassa (LASV), Machupo (MACV), Junin, Lujo, and lymphocytic choriomeningitis virus belong to the *Mammarenavirus*. LASV carries the largest disease burden, causing 300 000–500 000 infections per year in Western Africa. It is also the hemorrhagic fever most frequently exported out of Africa. The southeastern African genetically related counterpart of LASV is Mopeia virus (MOPV), a nonpathogenic virus [3]. The two viruses have a common rodent host (*Mastomys*), they cross‐react with polyclonal sera and share more than 75% amino acid identity, making MOPV an appropriate surrogate model. Arenaviruses are enveloped viruses containing a segmented, negative, and ambisense single‐stranded RNA genome. As an exception to the tri‐segmented *Antennavirus* genus, the viral RNA genome (vRNA) is comprised of two segments: a large segment (L) of around 7.2 kb and a small segment (S) of around 3.4 kb. Each segment uses an ambisense coding strategy to direct synthesis of two proteins in opposite orientation separated by an intergenic region (IGR). The L segment codes for the large protein L (~ 200 kDa), and a small RING protein Z (~ 11 kDa) that regulates replication and acts as the matrix protein of the virion. The S segment encodes for the multifunctional nucleoprotein (NP; see below) NP (~ 63 kDa) and the glycoprotein precursor (GPC; 75 kDa) that will eventually form the spikes at the surface of the virion. The IGR is thought to fold into secondary structures which lead to viral mRNA transcription termination. The RNA genome (and complementary) is always encapsulated in a polymer of NP forming the ribonucleoprotein complex (RNP).

The arenaviruses NP is the mandatory cofactor of the L protein for both transcription/replication processes, it protects passively and actively the genome/antigenome from cellular host defense enzymes, and finally, it packages the genome. These functions are reflected in the structural architecture of NP as a two domain protein surrounded by two flexible linkers : In amino terminal, the NP core domain (NP‐core) involved in polymerization and vRNA protection; in carboxy terminal, the exonuclease domain (ExoN) involved in degrading dsRNA, a marker of viral infection [4]. The sequence of NP is well conserved among arenaviruses (21% identity and ~ 80% similarity) and thoroughly along the NP structure (Fig. 1 & Fig. S1), which stressed the critical role of NP both at the structural and functional level. The first monomeric arenavirus NP crystallographic structure [5] confirmed its two domains architecture. It was puzzling though, because a NTP was captured in the RNA binding domain in a closed conformation. Further studies have observed that significant conformational changes are necessary to encapsidate the genomic RNA [6], and as part of the *Bunyavirales* and structural similarities with *Nairovirus*, arenaviruses NP should follow one of the four mechanism of multimerization [4]. Shortly, all *Bunyavirales* NP structures described until now, present a globular core domain, which, harbors the RNA binding cleft. From this core domain protrudes multimerization extensions either a single N‐ or C‐terminal, or both N‐ and C‐terminals, or else central α‐helices which are neither N‐ nor C‐terminal as in the case of *Nairovirus*. In contrast, the arenaviruses RNP structural data are limited to low‐resolution EM structure of Pichinde virus (PICV) RNP. The study by Young and Howard [7, 8] showed that PICV RNP is mostly formed by a flexible structure composed of NP monomers forming a filament. This filament appears to be a super helical structure, formed by intermediate helical structures composed of NP monomers associated with each turn of the helix in which the number of NP could not be resolved. They also observed smaller objects composed of two to three NPs. From more recent EM studies, trimeric assemblies were re‐observed and discussed in comparison with crystallographic data [9]. Nonetheless, this trimeric assembly does not account for RNA encapsidation.

**Fig. 1:**
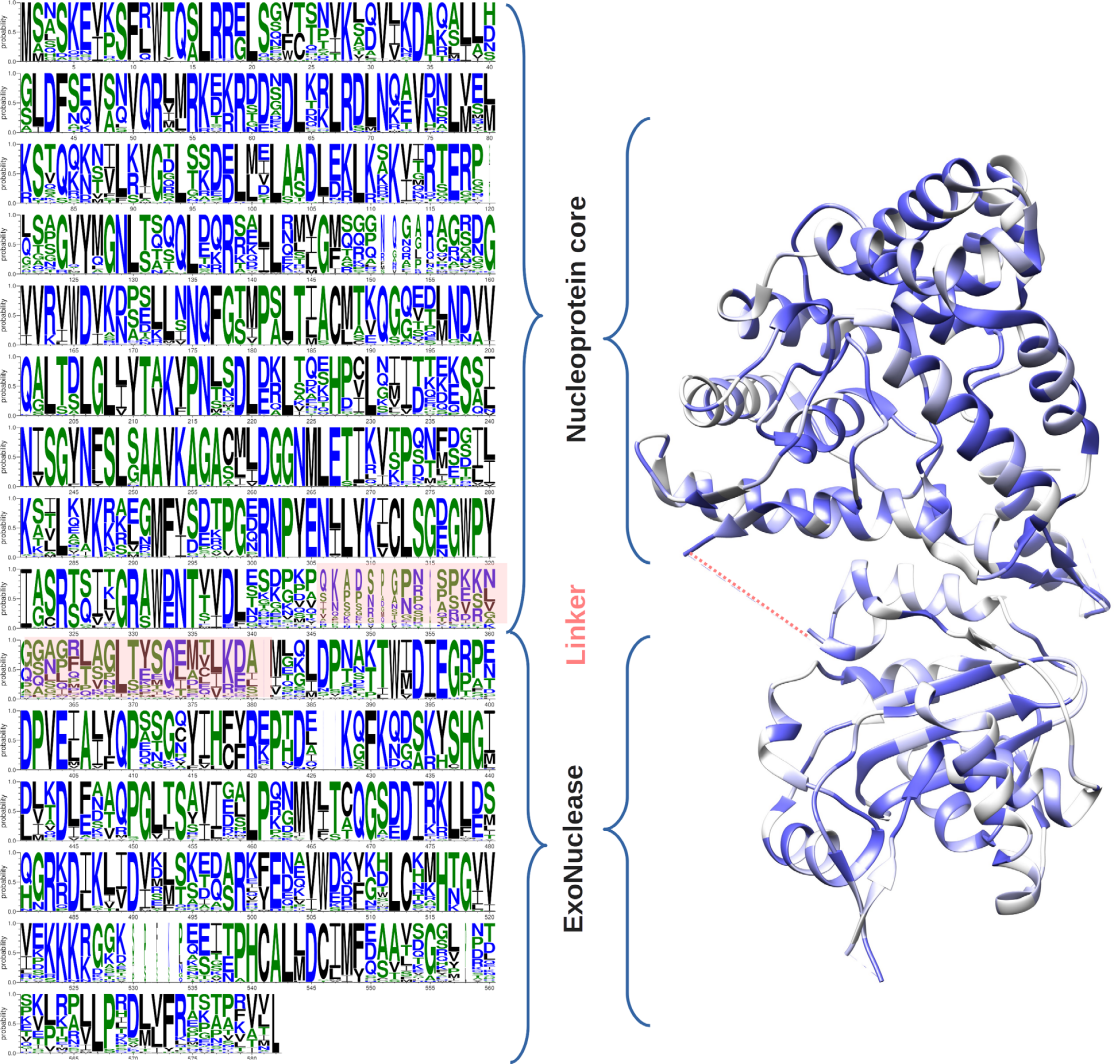
NP WebLogo representing the sequence conservation of *Mammarenavirus* sequences and their conservation on the structure (PDB: 3MWP). The WebLogo is derived from an alignment of 43 sequences (partial and identical sequences were previously removed). Domains and linker are indicated on the side of the alignment and the structure. Size of amino acid represents its conservation. On the structure, sequence identity is plotted by color change from deep blue (identical) to white (< 50%); missing linker is represented by a dashed line.

Here, we report a recent measurement of the full‐length MOPV NP by negative stain transmission electron microscopy (TEM). We observed MOPV RNP particles with diameters 15 ± 1 nm as well as circular heptamers of the same diameter, a result consistent with the original measurement of PICV RNP purified from the virus [7, 8]. We present a 3D reconstruction of these heptamers at 27 Å of resolution and discuss various structural characteristics.

## Materials and Methods

### Sequence alignment and sequence structure correlation analysis

*Mammarenavirus* NP sequences were retrieved from NCBI and uploaded in jalview [10]. All partial, incomplete, or identical sequences were discarded. Remaining sequences were aligned using mafft [11]. Sequence alignment is shown using WebLogo [12] and ESPRIPT [13], and the sequence conservation is visualized on the structure using UCSF CHIMERA [14].

### Protein production and purification

cDNA corresponding to MOPV‐NP was cloned by recombination (Gateway; Invitrogen) into pETG20A expression vector, which adds a cleavable N‐terminal thioredoxin‐hexahistidine tag. Protein was overexpressed in *Escherichia coli* strain C41(DE3) (Merck) grown in 2YT medium (Sigma‐Aldrich) at 37 °C to an OD600 _nm_ of 0.5. Expression was induced with 0.5 mm IPTG, and bacteria were grown shaking at 210 r.p.m. overnight at 17 °C in presence of 100 µm of ZnCl_2_. Bacteria were pelleted, frozen, and stored at −80 °C. The NP was purified by affinity chromatography using 2 mL of His pur^™^ cobalt column (Thermo Scientific; 20 mm Tris pH7.5, 300 NaCl, 5 mm imidazole, 0.5 m TCEP and eluted with the same buffer with 250 mm imidazole). The tag was removed by cleavage with TEV protease followed by a purification on a second cobalt affinity chromatography. Proteins were further purified by gel filtration using Superdex 75 column (GE Healthcare) in 20 mm Tris pH 7.5, 300 mm NaCl.

### Transmission electron microscopy and particle analysis

Transmission electron microscopy images were obtained from freshly purified full‐length MOPV‐NP at concentration 0.05 mg·mL^−1^. Drops of 5 μL were applied to a freshly deposited and glow‐discharged formvar‐carbon‐coated grid (Copper 300). The samples were stained with Nano‐W® (Nanoprobes) and transferred into a Tecnai 120 kV Electron Microscope. We have recorded 100 raw TEM images of 600 × 600 nm^2^ with an EAGLE 2k × 2k CCD camera. Images were under‐focused at 1–2 μm with a resolution of 2.8 Å/pix. Boxing, classification, initial model calculation as well as refinement for 3D reconstruction were done using the EMAN2 [15]. As NP is a 2 domains protein to assess the relative domain position, a model fitting and analysis were performed with CHIMERA. The fitting consists of a rigid body fit allowing shift and rotation using one single chain of the LASV‐NP structure (PDB: 3MWP) within the EM density map. To limit the fitting bias as well as to obtain quantitative information about the quality of the fit by a correlation coefficient, we have used the map calculated from the atoms of the 3MWP structure at resolution of 27 Å. Transformation of the pdb structure to a map was done by the program pdb2mrc included in the EMAN2 program.

## Results and Discussion

### Recombinant NP protein forms oligomers and can bind RNA

**Fig. 2:**
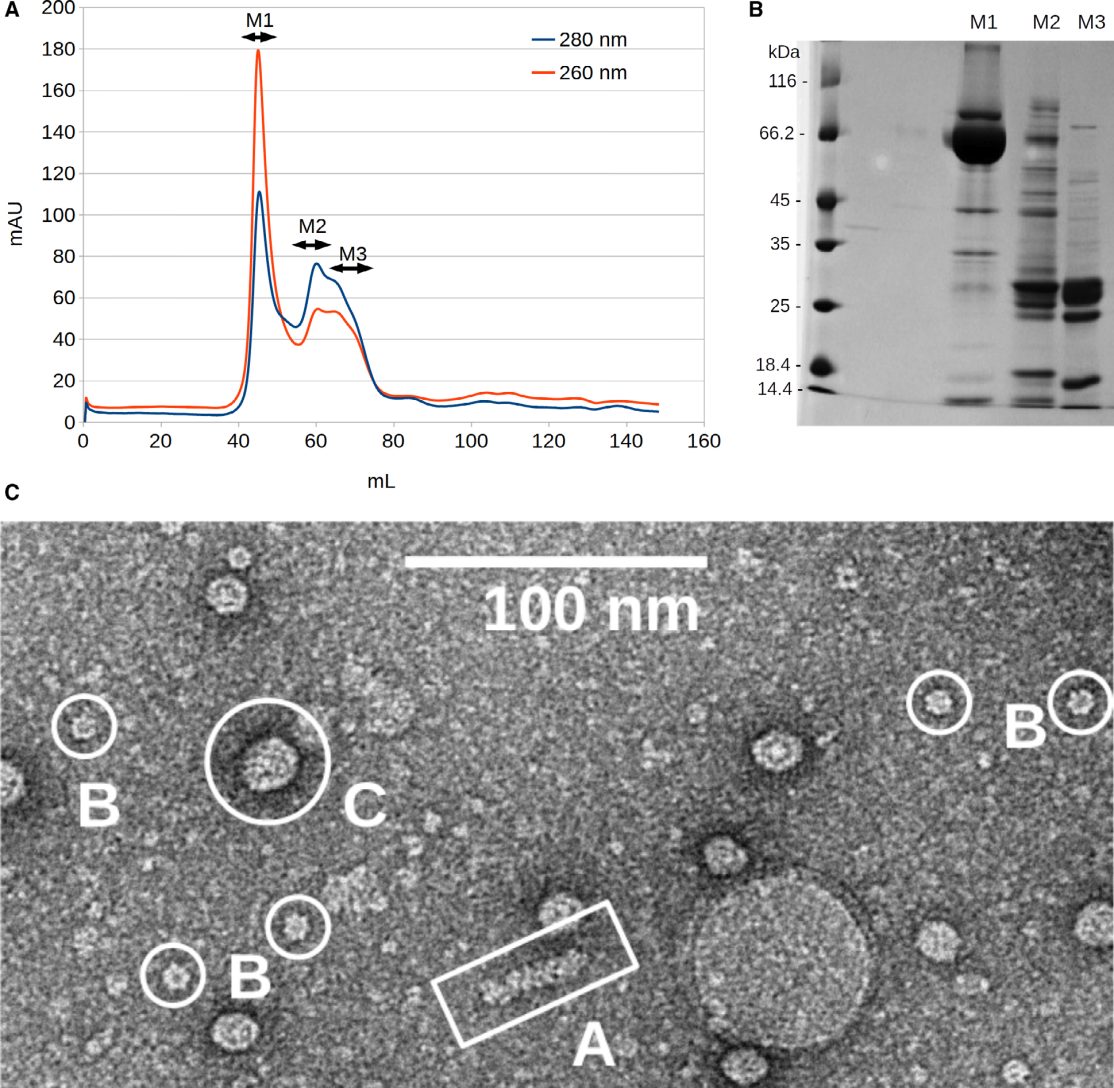
(A) MOPV‐NP size exclusion chromatography followed by UV at 280 nm (blue) and 260 nm (red). The three peaks fraction are delimited by two‐sided arrows and labeled M1, M2, M3. The M1 peak eluted as a multimer and has a 260/280 ratio over 1, indicating that the content is enriched in nucleic acid. (B) 12% SDS/PAGE analysis of M1, M2, M3 concentrated fractions, indicating that NP is mostly retrieved in M1. (C) General aspect of the sample studied shown in the bottom inset. The scale bar corresponds to a length of 100 nm.

MOPV‐NP was expressed in *E. coli* with a N‐terminal cleavable thioredoxin tag and purified it under nondenaturing conditions to preserve its structural integrity. The final gel filtration column shows three peaks, denoted as M1, M2, and M3 (Fig. 2, upper inset). SDS/PAGE analysis of concentrated samples reveals that M1 and M2 peaks contain a protein of the size expected for MOPV‐NP (~ 63 kDa). M1 corresponds to a higher mass assembly suggesting that NP was the main protein present in the sample (Fig. 2, upper inset). The position of peak M1 corresponds to a protein species with an apparent molecular mass of > 220 kDa, suggesting that NP forms higher‐order oligomers. In addition, the OD 260 nm/OD 280 nm ratio for the M1 peak fraction is of 1.57, clearly indicating that the higher‐order NP oligomers co‐elutes with nucleic acids, presumably RNA from the expression host.

**Fig. 3:**
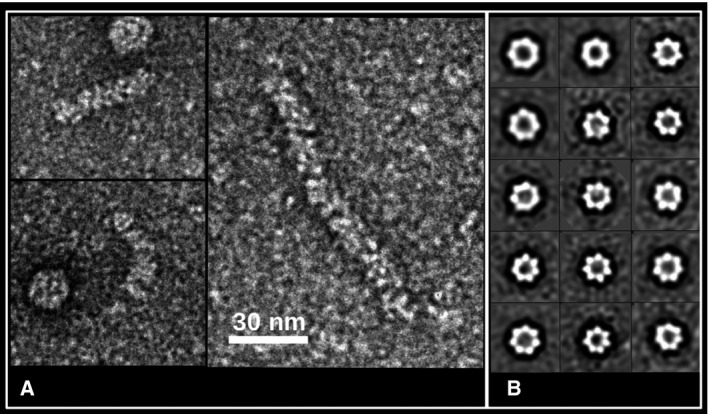
(A) TEM images of RNP particles from freshly purified MOPV‐NP protein. The scale bar show a length of 30nm (B) class averages corresponding to heptamers observed in this experiment.

In our TEM experiment, we have studied the M1 peak and observe three main types of particles shown in Fig. 2 (bottom inset): (a) few elongated RNP particles with typical diameters of 15 ± 1 nm indicated by the letter A. This observation is reminiscent to the PICV‐NP data previously observed with the presence of supercoiled structures forming fibers type structures of a diameter of 15 nm [7, 8]. The presence of RNP particles in our images suggests that the NP covers RNA segments. In Fig. 3A, a more detailed image of these RNP particles is shown in order to make clear their flexibility while the exact way of interconnection between NP monomers is not yet fully understood. (b) An abundance of circular multimers with typical diameter of 15 nm indicated by the letter B. The majority 80% of these particles presents a sevenfold symmetry while no lower symmetry multimers are observed, and finally (c) a great number of spherical particles with diameters ranging from 20 to 50 nm indicated by the letter C. The later may be formed by aggregation of NP monomers as they seem to be structured. The above observations demonstrate that MOPV‐NP is able to bind RNA and multimerize. Here, we speculate that there is a strong structural correlation between the observed isolated heptamers and the RNP particle relying on the following reasons: (a) The measured RNP filament width is comparable to the outer diameter of the isolated heptamers. (b) This organization is reminiscent of other *Bunyavirales* NPs. Indeed, it was previously observed in the case of Rift Valley Fever as well as Toscana virus [16, 17]. Negative stain EM studies on the NP of these previous virus have shown that the NP form in all cases multimers. Depending on the number of their monomers, some of these multimers stabilize in close ring‐shaped structures. On the other hand, their RNPs were constituted of helical turns featuring five to eight monomers. Let us note that the estimated number of monomers per turn represents an average value as the RNP is a helical structure and not a successive arrangement of circular rings. Moreover, the observed flexibility of the RNP is guaranteed by the possibility of local structural adaptation, driven by the local mechanical constraints which cannot be achieved by a fix number of monomers per turn. In general, the mean number of monomers per turn within the RNP filament is not a fix integer number. (c) As the observed diameter of PICV RNP filament is consistent with our own measurement, we propose that the isolated circular heptamers are quite representative of the average full turn of the Arenaviridae RNP filament.

### MOPV‐NP forms circular heptamers

**Fig. 4:**
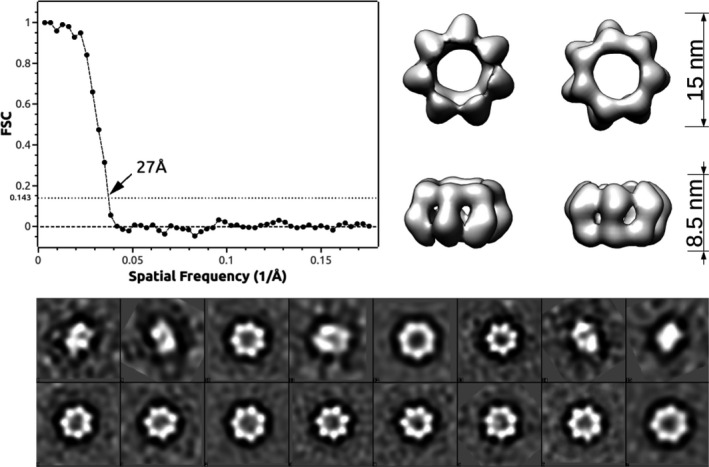
The upper part of the figure shows a 3D reconstruction of the observed heptameric particles at 27 Å resolution with the corresponding, FSC coefficient in function of the spatial frequencies in Å −1. At the bottom part of the figure are shown the particle classes used for the reconstruction.

In the following, we will focus on the circular heptamers shown in Fig. 3B. We collected 3000 particles corresponding to top, bottom, and side views of these sevenfold multimers. By 2D classification analysis, we kept 1225 particles corresponding to particle classes with higher contrast. An initial model was calculated with no symmetry restriction and subsequent refinement gave a 3D reconstruction of the observed multimer at a low resolution of 27 Å. For the refinement, a sevenfold symmetry axis was imposed (Fig. 4).

The 3D particle reconstruction was done by using the default EMAN2 pipeline. The result is shown in Fig. 4 together with the corresponding Fourier shell correlation (FSC) coefficient in function of the spatial frequencies in Å^−1^ units and the particle classes used in the refinement. The reconstructed particle has an external diameter of 15 nm an internal diameter of 5.3 nm and a height of 8.5 nm. The experimental error on all previous values is estimated at ± 1 nm.

**Fig. 5:**
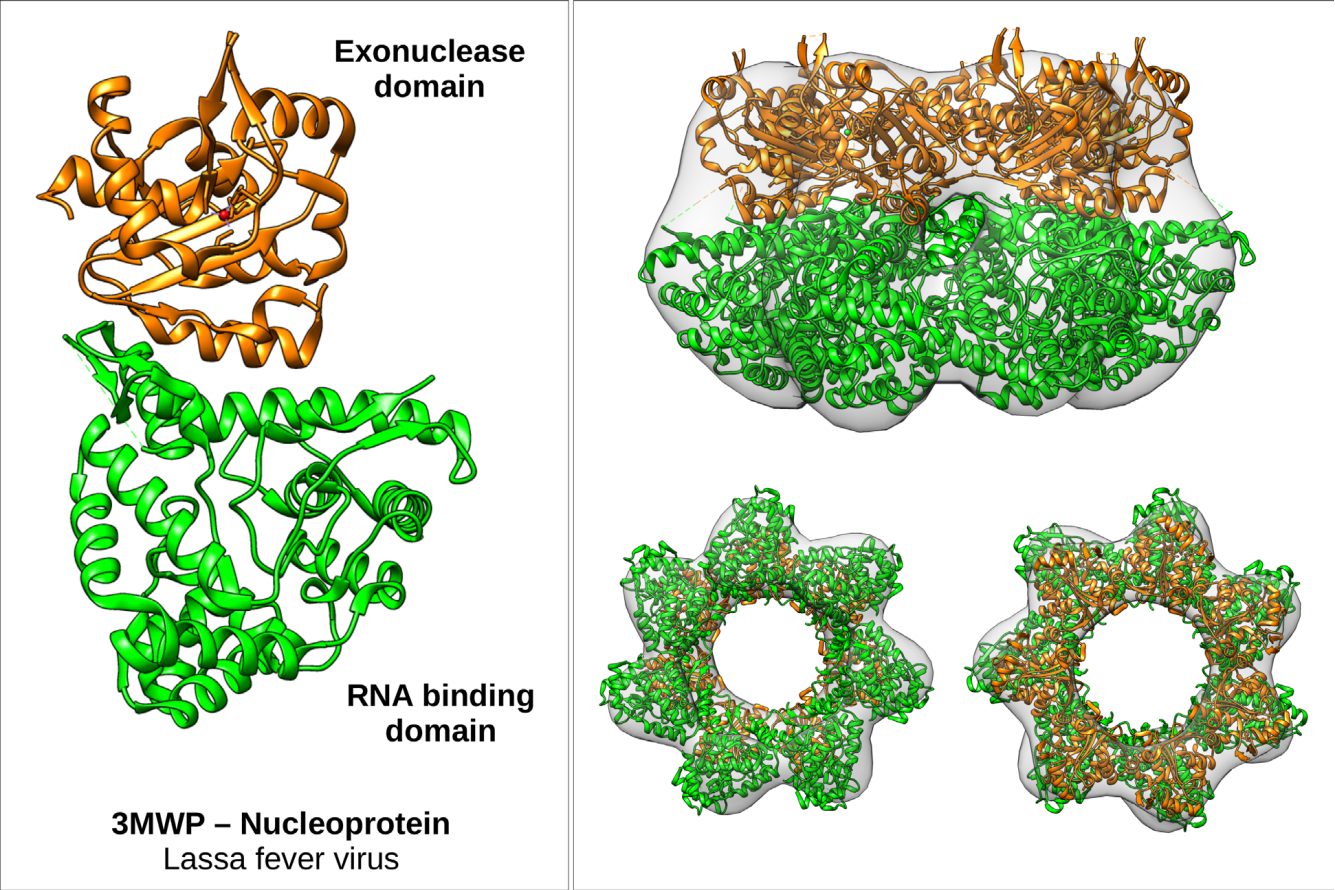
Volume analysis of NP density and comparison with crystallographic data.

Visual inspection of the 3D particle shows evidence of an elementary monomer containing two domains. Monomers are aligned and oriented in the same direction. In order to discuss a possible molecular model, we have considered here the structure of the LASV‐NP (PDB 3MWP) shown in Fig. 5 featuring two domains: The RNA binding domain (green) and the exonuclease domain (orange).

The length of the 3MWP molecule measured by CHIMERA from top to bottom is about 8 nm and can be nicely fitted inside the TEM 3D electron density. The fit was done as following: A single 3MWP model was first fitted in the 3D TEM density and used to generate symmetry partners following an arrangement with C7 symmetry imposed by the 3D electron density, as shown in Fig. 5. This heptamer model reproduces the diameter as well as the height of the measured 3D TEM electron density. The final heptamer model fit within the EM volume corresponds to a correlation coefficient CC of 0.88. Let us recall that for this fit we have transformed the pdb model structure to a map in order to have a quantitative information about the quality of the fit (CC).

However, this model is a rough approximation and it fails to give information about the exact multimerization mechanism between monomers. Moreover, the relative orientation between the exonuclease and the RNA binding domain results to a conformation where the RNA binding cavity is hidden by the exonuclease domain and no RNA covering can occur under these conformational conditions. More structural experiments are needed in order to elucidate this mechanism while the resolution of our TEM measurement does not permit to speculate further on the exact position of the exonuclease domain within the heptamer. However, this study brings evidence of a similar polymer assembly than of the other already characterized *Bunyavirales* NPs and questions the previously proposed assembly that NP assembles into a threefold symmetric complex [9].

In conclusion, this work reports the first observation of the complexed NP‐RNA as a polymer and hints its monomer assembly. The heptameric structure formed by the MOPV‐NP protein presented here shows that oligomerization occurs as it happens for other *Bunyavirales* NPs. However, our preliminary data do not allow to characterize how the multimerization is mediated between the subunits, yet this work is the first step toward high resolution of oligomeric NP characterization. In future experiments, we will bring high resolution of this RNP filaments and heptameric rings in order to characterize the structural elements that mediates the multimerization, using cryo‐EM approach. The use of standardized length RNA should help to stabilize this RNP structures and allow to define both the path that the genomic RNA is supposed to follow and the position of the exonuclease domain as well as if its catalytic site remains accessible to its substrate. Due to the central functions of NP to the virus life cycle, it remains critical to further characterize this polymer to identify potential sites that could be targeted for drug development.

## Conflict of interest

The authors declare no conflict of interest. The funders had no role in the design of the study; in the collection, analyses, or interpretation of data; in the writing of the manuscript, or in the decision to publish the results.

## Author contributions

NP and FF involved in conceptualization; AV, THVN, NP, and FF involved in methodology; AV, THVN, NP and FF made formal analysis; AV, THVN, NP and FF made investigation; NP and FF wrote original draft; AV, NP, FF, and BC wrote the review and edited the manuscript; NP and FF made visualization; NP and FF made supervision; FF administrated the project; FF and KA acquired the funding. All authors have read and agreed to the published version of the manuscript.

## Supporting information

Fig. S1. *Mammarenavirus* NP sequences alignment of 43 sequences used to generate the WebLogo.

## Data Availability

The data will be available from the corresponding author on reasonable request.
