# Supplementary material for: Observation of arenavirus nucleoprotein heptamer assembly
Source: FEBS Open Bio. 2021 Feb 25;11(4):1076–83. doi: 10.1002/2211-5463.13106 (PMC8016135; doi:10.1002/2211-5463.13106)

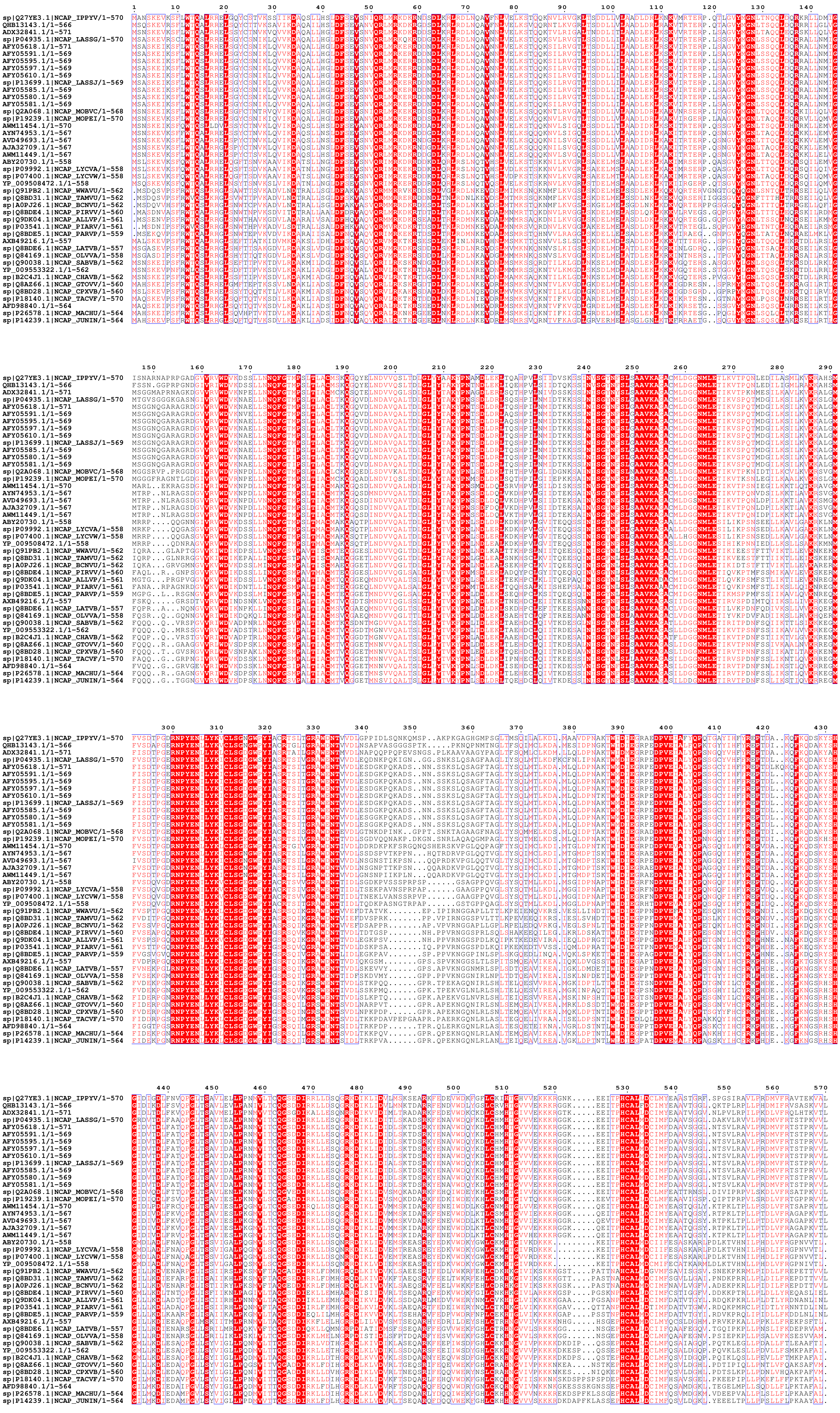

Supplement: Supplementary file 1 — Fig. S1. Mammarenavirus NP sequences alignment of 43 sequences used to generate the WebLogo. [file FEB4-11-1076-s001.pdf]
